# 150 years of rowing faster: what are the sources of more and more speed?

**DOI:** 10.1186/2052-1847-7-S1-O12

**Published:** 2015-08-11

**Authors:** Stephen Seiler

**Affiliations:** 1 Faculty of Health and Sport Sciences, University of Agder, Kristiansand, Norway

## 

Rowing has a 150 yr+ competitive history. Examining results from historic races like Oxford-Cambridge (established 1829) and the world championships (established 1893) reveals a linear increase in boat speed by 2-3% per decade. Boat velocity increases if propulsive power is increased and/or power losses are reduced. Over time, the propulsive power capacity of elite rowers has increased. Part of this increase is a result of recruiting athletes from a population that has become taller (1-3 cm per decade) and heavier. Modern world class rowers are typically 190-200 cm tall and weigh 90-100 kg. However, physical capacity does not scale directly with body dimensions but conforms instead to biological scaling laws. Increased rower mass also increases boat drag. Consequently, increased rower size only accounts for about 10% of the increase in boat speed. The tenfold increase in training load performed accounts for about 1/3 of the overall increase in physical capacity, and performance.

Power loss sources can be organized in terms of 1) boat drag, 2) oar blade inefficiency and 3) rowing technique. Racing rowing shells took on their modern form early, as boat design was revolutionized during the period 1830-1856. Boat weight reduction has been the only really significant development change since. Oar design and construction has evolved more slowly, but steadily, over time. The introduction of cleaver or “big” blades in 1991 exemplifies this evolution. Oar material and design improvements have decreased the loss of power to moving water by the blade. These changes probably account for ~25% of the overall improvement in boat velocity since 1856. Rowing technique as a potential source of inefficiency and power loss can be further subdivided into: 1) boat velocity fluctuations, 2) boat yaw, pitch, and roll, and 3) characteristics of the force curve during the rowing stroke. The now outlawed sliding rigger in the early 1980s demonstrated that decreasing boat velocity fluctuations improved racing performance. Boat yaw, pitch, and roll varies across rowers and is a potential source of power loss due to both direct effects on boat drag and indirect effects on power production. However, the best rowers are able to row with vary minor imbalance in the boat. Force curve optimization is heavily discussed today. Real time measurement of boat kinematics and rower force application patterns open for new approaches to training and rower selection for team boats. It seems unlikely that one optimal force curve can be identified for all rowers in a team boat because the interaction among anatomical, muscular, and biomechanical factors probably constrains the optimal force curve for each rower.

